# Recurrent intra-mural esophageal abscess as a complication of untreated eosinophilic esophagitis

**DOI:** 10.1055/a-2621-2827

**Published:** 2025-08-14

**Authors:** Marianne Hupé, Amaury DʼAngelo, Arthur Berger, Frank Zerbib

**Affiliations:** 1 36724 University of Grenoble Alpes/Hepato-Gastroenterology and Digestive Oncology Department, CHU Grenoble Alpes/Institute for Advanced Biosciences, Grenoble, France; 2 36836 CHU de Bordeaux, Centre Médico-chirurgical Magellan, Hôpital Haut-Lévêque, Department of Gastroenterology, Université de Bordeaux, INSERM CIC 1401, Bordeaux, France

Eosinophilic esophagitis (EoE) is a condition characterized by abnormal infiltration of the esophageal mucosa with eosinophils, leading to symptoms such as dysphagia. Common complications, often seen in undertreated cases, include fibrosis-related issues such as food impaction and strictures 1 . We present the case of a patient born in 1999 who, in spring 2021, was admitted with septic dysphagia, leading to the diagnosis of an intramural esophageal abscess. Endoscopy revealed a mucosal tear located 25 cm from the superior dental arch (SDA), while histology confirmed esophageal eosinophilic infiltration exceeding 20 eosinophils per high-power field (HPF). The abscess was resolved with antibiotic therapy, and the patient was prescribed proton pump inhibitors (PPIs). However, due to poor treatment compliance, subsequent gastroscopies revealed a stricture, which required dilation in September 2021.

In March 2022, the patient was readmitted for recurrent septic dysphagia. A computed tomography scan revealed another esophageal abscess. Endoscopy ( Video 1 ) showed a secondary opening at 30 cm from the SDA, overlying a parietal bulge extending to 36 cm ( Fig. 1 ). Spontaneous pus drainage was facilitated using a Hook knife, followed by irrigation for cavity cleansing ( Fig. 2 ).

**Fig. 1:**
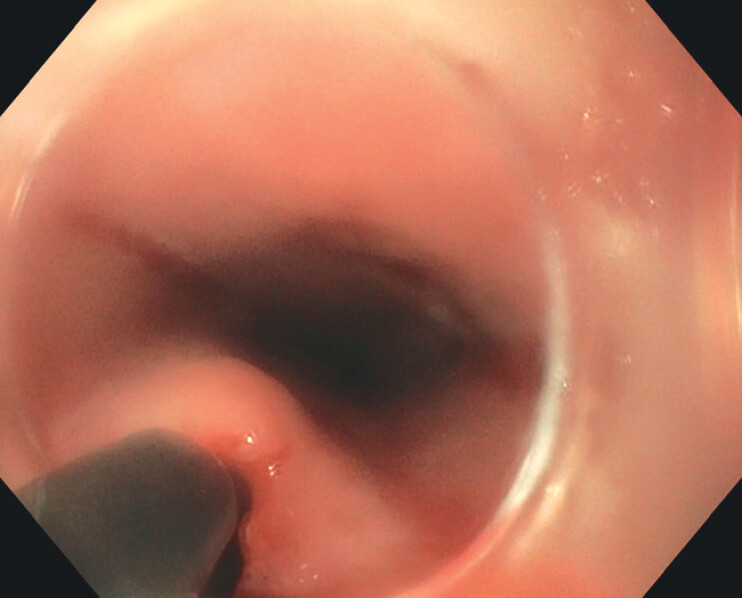
Endoscopic drainage of intramural abscess related to eosinophilic esophagitis.

**Fig. 2:**
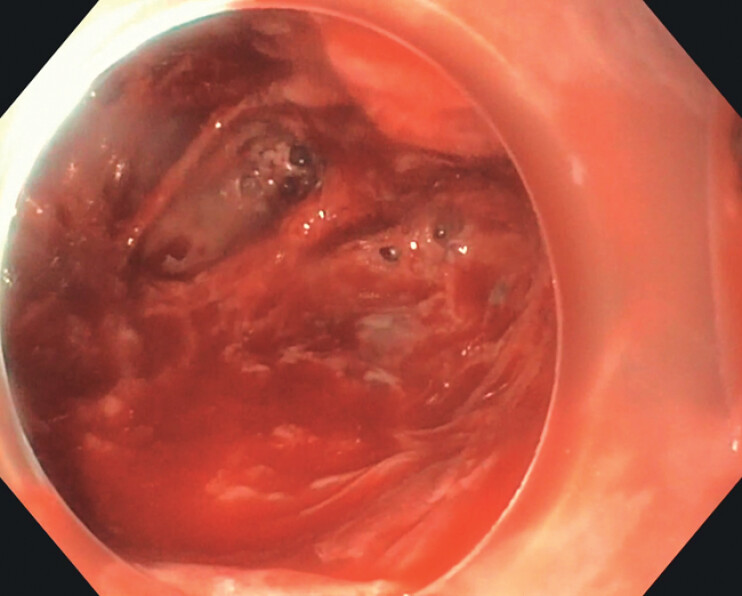
Intramural cavity aspect after endoscopic drainage.

Endoscopic view of an intramural esophageal abscess, with commentary, before and after drainage using a Hook knife.Video 1

By May 2022, the patient was asymptomatic and compliant with PPI therapy; however, endoscopy revealed esophageal rings, and histology showed persistent eosinophilic infiltration. His treatment was switched to orodispersible budesonide.

In July 2022, the patient presented again with dysphagia. Endoscopy revealed grade C esophagitis and an aggravating stricture, which was dilated to 17 mm using a bougie. Combination therapy with PPIs and budesonide was initiated. A follow-up in September 2022 showed a resolution of esophagitis and a decrease in eosinophilic infiltration to nine HPF. The patient remains asymptomatic.

This case highlights the complexity of managing EoE, a condition potentially linked to reflux, and illustrates a rare complication underscoring the importance of achieving histological remission.

Endoscopy_UCTN_Code_CCL_1AB_2AC

## Notes

Endoscopy E-Videos https://eref.thieme.de/e-videos
*E-Videos* is an open access online section of the journal *Endoscopy* , reporting on interesting cases and new techniques in gastroenterological endoscopy. All papers include a high-quality video and are published with a Creative Commons CC-BY license. Endoscopy E-Videos qualify for HINARI discounts and waivers and eligibility is automatically checked during the submission process. We grant 100% waivers to articles whose corresponding authors are based in Group A countries and 50% waivers to those who are based in Group B countries as classified by Research4Life (see: https://www.research4life.org/access/eligibility/ ). This section has its own submission website at https://mc.manuscriptcentral.com/e-videos .
